# Proprioceptive Interaction between the Two Arms in a Single-Arm Pointing Task

**DOI:** 10.1371/journal.pone.0137031

**Published:** 2015-08-28

**Authors:** Kazuyoshi Kigawa, Masahiko Izumizaki, Setsuro Tsukada, Naoyuki Hakuta

**Affiliations:** 1 Department of Physiology, Showa University School of Medicine, Tokyo, Japan; 2 Department of Orthopaedic Surgery, Showa University Fujigaoka Hospital, Yokohama, Japan; 3 Department of Neurology, Showa University School of Medicine, Tokyo, Japan; Birkbeck, University of London, UNITED KINGDOM

## Abstract

Proprioceptive signals coming from both arms are used to determine the perceived position of one arm in a two-arm matching task. Here, we examined whether the perceived position of one arm is affected by proprioceptive signals from the other arm in a one-arm pointing task in which participants specified the perceived position of an unseen reference arm with an indicator paddle. Both arms were hidden from the participant’s view throughout the study. In Experiment 1, with both arms placed in front of the body, the participants received 70–80 Hz vibration to the elbow flexors of the reference arm (= right arm) to induce the illusion of elbow extension. This extension illusion was compared with that when the left arm elbow flexors were vibrated or not. The degree of the vibration-induced extension illusion of the right arm was reduced in the presence of left arm vibration. In Experiment 2, we found that this kinesthetic interaction between the two arms did not occur when the left arm was vibrated in an abducted position. In Experiment 3, the vibration-induced extension illusion of one arm was fully developed when this arm was placed at an abducted position, indicating that the brain receives increased proprioceptive input from a vibrated arm even if the arm was abducted. Our results suggest that proprioceptive interaction between the two arms occurs in a one-arm pointing task when the two arms are aligned with one another. The position sense of one arm measured using a pointer appears to include the influences of incoming information from the other arm when both arms were placed in front of the body and parallel to one another.

## Introduction

The awareness of the positions of the body’s limbs occurs not only through vision but also through proprioception [1]. There is some evidence that proprioceptive signals coming from both arms are used to determine the perceived position and movement of one arm [2–5]. The perceived position of one arm may represent a difference signal calculated using the input coming from both arms [6, 7]. We have shown that when both arms are vertically aligned and then one arm is moved into an extension, the perception of a slower vibration-induced illusion occurs in the other arm [3]. Because elbow extension increases the spindle signal in its own elbow flexors, the difference in spindle discharge rate between the two arms is reduced. What is perceived during the extension illusion of one arm is based, at least in part, on a difference signal derived from the inputs of both arms [7].

The sense of limb position can be measured in the arms using a two-arm matching task [6]. Typically, an experimenter places the participant’s unseen reference arm at a particular elbow angle, and the participant is asked to match the angle by placement of the other arm [2, 3, 8–14]. Combined with the two-arm matching task, externally applied muscle vibration of the reference arm has been used in studies investigating the role of muscle spindle activity in proprioceptive sensation [2, 3, 9, 10, 12–14]. An important characteristic of muscle vibration is its ability to selectively excite the primary endings of muscle spindles [15] and cause vibration illusion [16]. To give an example of vibration illusion, muscle vibration of elbow flexors produces an illusion of changed position in the direction of elbow extension [6]. Recently, we have shown that vibration illusion of the reference arm was reduced by muscle vibration of the other arm in a two-arm matching task [2]. This observation highlights the importance of the difference signal in the position sense between the two arms in a two-arm matching task.

The limb position sense can also be measured using a one-arm pointing task [17, 18]. In the one-arm pointing task, participants indicate the position of the unseen reference arm with a pointer, not with the other arm. The pointer is a part of a mechanical device, not a body part. Thus, an important difference between the matching and pointing tasks is that an afferent signal from the pointer is unavailable in the one-arm pointing task to specify the position of the reference arm. Alternatively, it has been speculated that in pointing tasks reference is made to a central map of the body, the postural schema, to locate the position of the reference arm [6, 7, 19]. Stored body representations have been shown by various means [19–24]. For example, Longo and Haggard [19] provided evidence in support of a stored model of the body’s metric properties. Their subjects used a long metal baton with their right hands to point to the position of 10 landmarks on their unseen left hands. The subjects successfully drew a central body representation of their hands, which included distortions in size and shape of the hand. The postural schema is referred to as a perceptual representation of the posture of the body, but this pre-existing representation in the brain is plastic and continuously updated by proprioceptive afferent information [25, 26].

We previously raised the possibility that what appears to be a one-arm proprioceptive test (i.e., the pointing task) may in fact include some input from the other arm [3]; however, verification of this hypothesis awaited further investigation. We also speculated that the interaction in the position sense between the two arms, reported in a two-arm matching task [2, 3], occurs only when the two arms are aligned to act as a single instrument [2, 7]. Here, we examined whether the perceived position of one arm is affected by proprioceptive signals from the other arm in a one-arm pointing task. First of all, we examined whether proprioceptive illusions elicited by the vibration of elbow flexors in the reference arm were altered by increases in the muscle spindle signals in the other arm in a one-arm pointing task (Experiment 1). If we determined that proprioceptive interaction between the two arms occurred in the one-arm pointing task, we then planned to test whether the interaction occurred irrespective of arm position (Experiment 2). Thus, we could determine whether the position sense of the reference arm was affected by proprioceptive signals coming from the other arm placed at an irrelevant position. In Experiment 3, we planned to extend the findings of Experiment 2 by examining whether the vibration-induced extension illusion of the reference arm occurred to the same extent regardless of the position of this arm. The overall aim of the work was to examine whether the perceived position of the reference arm is affected by proprioceptive signals from the other arm in a one-arm pointing task. We found that proprioceptive interaction between the two arms occurred in this task when the two arms were aligned with one another.

## Materials and Methods

### Participants

A total of 15 male students participated in this study, which consisted of Experiments 1, 2, and 3, with 10 volunteers participating in each experiment. Five volunteers participated in Experiments 1, 2, and 3, two in Experiments 1 and 2, two in Experiments 2 and 3, one in Experiments 1 and 3, and five in one of the three experiments. All participants included in this study gave their written, informed consent before undertaking the experiments, which were approved by the Showa University Committee for Human Experimentation. Ethical aspects of the experiments conformed to the 1964 Helsinki declaration and its later amendments.

### Testing apparatus

**Fig 1:**
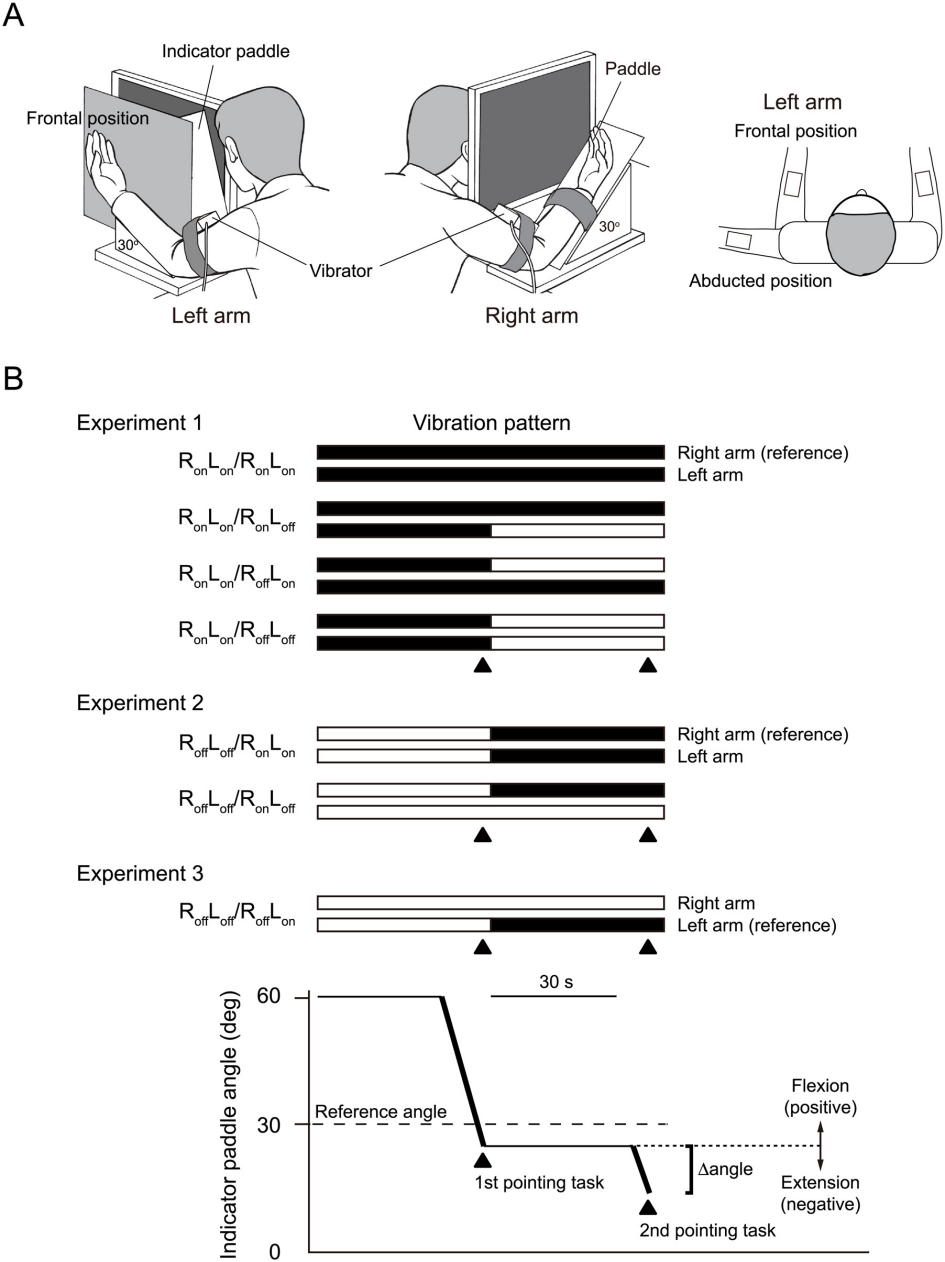
Apparatus and protocol. (A) The device has two paddles. The participants’ right forearm was strapped to the right paddle and set at an angle 30° from the horizontal. The left forearm was placed on another support block at a 30° angle. In Experiment 1, the left arm was always placed in front of the body. In Experiments 2 and 3, the left arm was placed in front of the body or at an abducted position. Each vibrator was placed on the distal tendon of the biceps of each arm. The left paddle (indicator paddle) was used as an indicator that measured the perceived vertical angle of the unseen reference forearm to the horizontal surface. (B) Vibration patterns during pointing tasks. Filled bars represent the presence of vibration, and open bars represent its absence. The actual angle of the reference forearm was always 30° from the horizontal (dashed line). The perceived angle of the reference forearm was measured twice in each trial: the first and the second pointing tasks (filled triangles). The differences between the angles obtained in the first pointing task (dotted line) and the second pointing task were calculated for each of the four conditions (angle difference; Δangle). When the indicator paddle was placed in a more extended position in the second pointing task than in the first pointing task, Δangle was expressed as a negative value. If the indicator paddle was placed in a more flexed position, Δangle was expressed as a positive value.

The perceived position of the unseen forearm was specified by the participant with an indicator in the vertical plane using a tabletop, custom-built device (Fig 1A) [17]. The device consisted of two paddles, one on the right and one on the left side. The participant’s right forearm was strapped to the right paddle and placed at an angle of 30° from the horizontal, with a support block under the paddle. The left forearm was placed on another support block, also inclined at 30°. The left paddle (indicator paddle) was used as an indicator that measured the perceived position of the reference arm. The reference arm was the right arm in Experiments 1 and 2 and the left arm in Experiment 3. The paddle hinge was arranged to coincide with the elbow joints. There was one partition between the right arm and the indicator paddle and another between the indicator paddle and the left arm, arranged to block the participants’ views of their own right and left arms, but allowing them to see the indicator paddle. In Experiments 2 and 3, the left arm was placed in front of the body and parallel to the right arm, or it was rotated externally (abducted position) (Fig 1A, right panel). The participant directed an experimenter to move the indicator paddle to match the position of the unseen reference arm.

### Measurement of vertical angles

The angle of the indicator paddle was measured relative to the horizontal. A rotary encoder (E6H-CWZ6C, OMRON, Kyoto, Japan) was fixed to the side of the paddle. The encoder provided a pulse output every 0.1° above the horizontal (0°). The number of pulses was counted by a pulse meter (K3HB-C, OMRON) that sent a linear output signal to a recording device (Power Lab, 4/20, ADInstruments, Castle Hill, NSW, Australia). All data were recorded using LabChart software (ADInstruments) with a computer.

### Vibrators

Two custom-made vibrators were used in this study. Each vibrator comprised a motor-driven eccentric disk that was enclosed in a plastic box measuring 50 mm × 60 mm × 88 mm. The vibration amplitude was approximately 1 mm and the frequency could be adjusted between 70 and 80 Hz, close to the optimum for engaging responses from muscle spindles [15]. Each vibrator was placed on the distal tendon of the biceps of each arm using elastic straps (Fig 1A).

### Muscle conditioning before matching

Muscle conditioning was conduced before each pointing task. Before each trial, both arms were conditioned to place them in a defined and comparable mechanical state [27, 28]. To do this, the elbow flexors of both arms, which were aligned with one another in front of the body, were first contracted at the vertical angle of 30° because muscle shortening or lengthening after contraction changes the muscle spindle activity, leading to position sense illusions as a result of muscle thixotropy [13, 27]. For this conditioning, the participant was asked to flex each arm with a contraction of perceived 30–50% of maximum for 3 s while the experimenter held the participant’s wrists; the participant was then asked to relax. Experiments were started within 30 s after conditioning.

### Experiment 1

The aim of this experiment was to examine whether muscle vibration of one arm affected the position sense of the other arm in the vertical plane using a one-arm pointing task (*n* = 10) (Fig 1A). The right arm was used as the reference arm. Thus, we examined whether the presence and absence of left arm vibration changed the extension illusion in the right arm. Both arms were placed in front of the body and parallel to one another throughout Experiment 1.

Participants had their eyes open and were able to see the indicator paddle, but they could see neither their right nor left arms because their own arms were hidden from view by the partitions (Fig 1A). A schema for Experiment 1 is shown in Fig 1B. Both arms were continuously maintained at an angle 30° from the horizontal (Fig 1A). After participants completed conditioning both arms, vibration of elbow flexors in both arms commenced (Fig 1B). After a 30-s vibration period, participants were asked to specify the angle of the right arm using the indicator paddle while vibration of the elbow flexors in both arms continued. An experimenter placed the indicator paddle at 60° and then began moving the paddle toward the horizontal plane. The experimenter changed the speed of the movement or the angle of the paddle if requested by the participant. When the participants declared that the angle of the indicator paddle matched that of the right arm, the angle of the indicator paddle was measured. This was referred to as the first pointing task.

Thirty seconds after completion of the first pointing task, the second pointing task was conducted under each of four testing conditions, with and without vibration of the elbow flexors of the right and left arms (Fig 1B). The indicator paddle was maintained at the angle determined by the participant in the first pointing task until the start of the second pointing task. When vibration was continued in both arms after the completion of the first pointing task, this condition was referred to as R_on_L_on_/R_on_L_on_. The R_on_L_on_ before the slash denotes vibrating the right and left arms during the first pointing task, and the R_on_L_on_ after the slash indicates vibrating the right and left arms for the second pointing task. For the R_on_L_on_/R_on_L_off_ condition, the vibrator of the left arm was turned off after completion of the first pointing task, but the vibrator of the right arm remained on until completion of the second pointing task. For the R_on_L_on_/R_off_L_on_ condition, the vibrator of the right arm was turned off after completion of the first pointing task. For the R_on_L_on_/R_off_L_off_ condition, both vibrators were tuned off after completion of the first pointing task. The different conditions were presented in random order, and each condition was repeated three times for a total of 12 trials with each participant.

### Experiment 2

This experiment tested the hypothesis that muscle vibration of the other arm alters the vibration-induced illusion of elbow extension in the reference arm when both arms are aligned in front of the body, but not when the other arm is in an abducted position (Fig 1A, right panel). The right arm was used as the reference arm in this experiment.

Participants could see the indicator paddle but not their own arms (Fig 1A, left and middle panels). The right arm was continuously maintained in front of the body at an angle of 30° from the horizontal, as in Experiment 1 (Fig 1A, middle panel). The left arm was also maintained at a 30° angle but was placed either in front of the body parallel to the right arm (frontal position) or at an externally rotated position perpendicular to the right arm (abducted position) (Fig 1A, right panel).

Conditioning muscle contractions were conducted in both arms as described above for Experiment 1. The pointing accuracy was then measured when neither arm was vibrated (Fig 1B, first pointing task). The perceived angle of the right arm—the actual angle of this arm was always 30°—was measured using the indicator paddle by an experimenter who moved the indicator paddle from 60° toward the horizontal plane, stopping when the participant declared a match between the angles of the indicator paddle and the right arm. The experimenter changed the speed of the movement and angle of the paddle if requested by the participant.

The second pointing task for this experiment was conducted under each of four testing conditions, with or without vibration of the elbow flexors of the left arm placed in the frontal or abducted positions. Under the R_off_L_off_/R_on_L_on_ condition, elbow flexors of both arms were vibrated after the first pointing task. The left arm was placed in the frontal or abducted position. After a 30-s vibration period, participants were asked to specify the angle of their vibrated right arm, and an experimenter moved the indicator paddle to match the perceived position. Under R_off_L_off_/R_on_L_off_ conditions, elbow flexors of the right arm were vibrated after the first pointing task, while those of the left arm were not. The four conditions were as follows: (1) R_off_L_off_/R_on_L_on_, with the left arm at the frontal position; (2) R_off_L_off_/R_on_L_on_, with the left arm at an abducted position; (3) R_off_L_off_/R_on_L_off_, with the left arm at the frontal position; (4) R_off_L_off_/R_on_L_off_, with the left arm at an abducted position. The conditions were presented in random order, and each condition was repeated three times, for a total of 12 trials in each participant.

### Experiment 3

The purpose of this experiment was to test whether the vibration-induced position sense illusion of one arm was altered by the position of the same arm. Specifically, the goal was to determine whether the brain received fewer spindle signals from a vibrated arm placed at an abducted position than in front of the body. Different from Experiments 1 and 2, the left arm was used as the reference arm in Experiment 3. Participants specified the perceived position of the unseen left arm with the indicator paddle. The left arm was placed in front of the body and parallel to the right arm (frontal position) or at an externally rotated position perpendicular to the right arm (abducted position) at an angle 30° from horizontal and resting on a support block (Fig 1A, right panel).

Conditioning muscle contractions were conducted in both elbow flexors in the test position prior to the start of this experiment as described above in Experiment 1. Pointing accuracy was measured before the start of vibration (Fig 1B, first pointing task). The perceived angle of the left arm—the actual angle was always 30° from the horizontal—was measured using the indicator paddle. During the first pointing task in Experiment 3, an experimenter moved the indicator paddle from 60° toward the horizontal plane until the participant declared a match between the angles of the indicator paddle and the left arm. After completion of the first pointing task, the vibration of the left arm began. The right arm was not vibrated throughout this experiment. The experimenter set the indicator paddle at the angle specified by the participant during the first pointing task. After a 30-s vibration period, participants were asked to match the angle of the indicator paddle with that of the left arm. When the participants declared that the angle of the indicator paddle matched the perceived position of the left arm, the angle was measured (second pointing task). The vibration continued until the completion of the second pointing task. The two positions of the left arm (frontal or abducted position) were used in random order, and each position was repeated three times for a total of six trials in each participant.

### Statistical analysis

The differences were calculated between the angles obtained in the first and second pointing tasks in each experiment (angle difference, Δangle) (Fig 1B, bottom panel), and the mean for the three repetitions in a single participant was used as the value representing that participant. The Δangle was assumed to reflect the effect of each condition on the sense of position because the angles in each of the first pointing tasks were measured under identical conditions for each experiment. Commercially available software packages were used for the analysis (Prism 6; GraphPad Software Inc., La Jolla, CA, USA, and IBM SPSS Statistics 21; SPSS Inc., Chicago, IL, USA). Values of *P* < 0.05 were considered statistically significant. Values are presented as means ± SEM.

## Results

Throughout Experiment 1, both arms were aligned with one another in front of the body. The reference arm was the right arm. Elbow flexors of both arms were vibrated during the first pointing task (R_on_L_on_/). The second pointing task was performed subsequently with different combinations of the right arm vibration and the left arm vibration (/R_on_L_on_, /R_on_L_off_, /R_off_L_on_, /R_off_L_off_). These tasks made for four different conditions: R_on_L_on_ /R_on_L_on_, R_on_L_on_ /R_on_L_off_, R_on_L_on_ /R_off_L_on_, R_on_L_on_ /R_off_L_off_. In Experiment 2, the right arm was the reference arm and always placed in front of the body, while the left arm was placed at a frontal position or at an abducted position. The first pointing task was performed when neither arm was vibrated (R_off_L_off_/). The second pointing task was performed with both arms vibrated (/R_on_L_on_) or with the right arm vibrated (/R_on_L_off_). Combinations of vibration patterns and the left arm position made for four different conditions: R_off_L_off_/R_on_L_on_ with the left arm placed at a frontal position, R_off_L_off_/R_on_L_on_ with the left arm placed at an abducted position, R_off_L_off_/R_on_L_off_ with the left arm placed at a frontal position, R_off_L_off_/R_on_L_off_ with the left arm placed at an abducted position. In Experiment 3, note that the left arm was the reference arm, and it was placed at a frontal position or at an abducted position. The right arm was always placed in front of the body. The first pointing task was performed when neither arm was vibrated (R_off_L_off_/). The second pointing task was then performed with the left arm vibrated (/R_off_L_on_). These made for two different conditions: R_off_L_off_/ R_off_L_on_ with the left arm placed at a frontal position, and R_off_L_off_/ R_off_L_on_ with the left arm placed at an abducted position.

### Experiment 1

**Fig 2:**
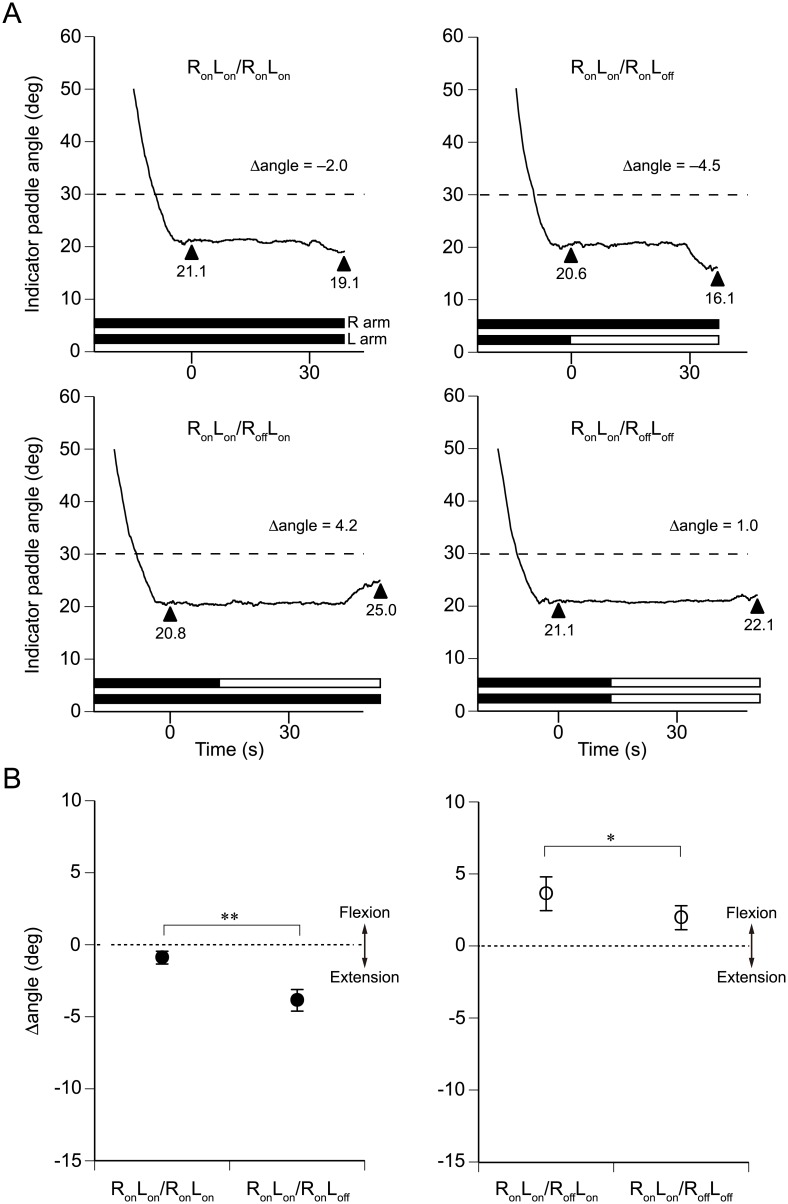
Reference arm pointing errors during vibration of reference and non-reference arms. (A) Each panel represents a single arm pointing trial in a single participant in Experiment 1. The continuous line in each panel indicates the position of the indicator paddle. Left triangles represent the angle of the perceived position of the reference arm in the 1st pointing task, and right triangles represent the angle in the second pointing task. Filled bars represent the presence of vibration, and open bars represent its absence. An angle of 0° represents the horizontal position. The position of the reference forearm was always at 30° (dashed lines). Time zero represents the time when the participant declared that the angle of the indicator paddle matched that of the right arm in the 1st pointing task. (B) Pooled data for the group (*n* = 10). In the left panel, the illusion of elbow extension induced by vibrating the right arm elbow flexors was increased more when the left arm vibration was turned off (R_on_L_on_/R_on_L_off_) than when it was continued (R_on_L_on_/R_on_L_on_) (**P < 0.01). In the right panel, when the left vibration was continued while the right arm vibration was turned off (R_on_L_on_/R_off_L_on_), the vibration-induced illusion of elbow extension, which had been induced by the preceding vibration of elbow flexors of the right arm, withdrew more than when both right arm vibration and left arm vibration were turned off (R_on_L_on_/R_off_L_off_) (*P < 0.05). Values are means ± SEM.

Traces from a representative participant are shown in Fig 2A. Each panel shows one of three trials for each of the four tested conditions. The upper two panels of Fig 2A represent trials performed when the reference (right) arm was vibrated until the completion of the second pointing task. When both arms were vibrated for the first pointing task in the R_on_L_on_/R_on_L_on_ condition (Fig 2A, upper left panel), the pointing angle was 21.1° from the horizontal plane. Thus, the participant perceived his unseen reference arm to be more extended than it actually was. Vibration of both arms was continued for the second pointing task. After another 30-s vibration period, the participant was again asked to match the angle of the indicator paddle with that of the reference arm. The participant declared a match at 19.1° (Fig 2A, upper left panel). As a consequence, an extended period of vibration of both arms caused the Δangle to be −2.0° (19.1°–21.1°). For the R_on_L_on_/R_on_L_off_ condition, the left arm vibration was turned off after the first pointing task (Fig 2A, upper right panel). As a result, the indicator paddle showed an extension from 20.6° to 16.1° (Δangle = −4.5°). For this individual, a comparison between the Δangles of –2.0° and –4.5° suggested that the left arm vibration decreased the angle of the extension illusion in the vibrated reference arm by 2.5°.

Using the R_on_L_on_/R_off_L_on_ and R_on_L_on_/R_off_L_off_ conditions, the pointing task was repeated in the same participant but with the reference (right) arm vibration turned off after the first pointing task (Fig 2A, lower two panels). When the reference arm vibration was turned off, the angle of the perceived position increased toward the actual reference angle of 30°. For the second pointing task, the participant felt the reference arm to be less extended in the presence of vibration of the other arm (Fig 2A, left panel, 25.0°, Δangle = 4.2°) than in the absence of vibration of the other arm (Fig 2A, right panel, 22.1°, Δangle = 1.0°). For this individual, this meant that vibration of the other arm decreased the position sense error of the reference arm by 3.2°. It was also noted that the indicator paddle was still placed at an extended position even though the reference arm vibration was stopped before the second pointing task (Fig 2A, lower two panels).

The pooled data for the 10 participants confirmed the results for the individuals (Fig 2B). The mean values of the indicator paddle angle for the first pointing task were similar: 17.3° ± 0.8° (R_on_L_on_/R_on_L_on_ condition), 17.1° ± 0.8° (R_on_L_on_/R_on_L_off_ condition), 16.4° ± 1.1° (R_on_L_on_/R_off_L_on_ condition), and 17.1° ± 1.2° (R_on_L_on_/R_off_L_off_ condition). The mean values for the Δangle under the conditions of R_on_L_on_/R_on_L_on_ and R_on_L_on_/R_on_L_off_ were −0.9° ± 0.4° and −3.9° ± 0.8°, respectively (Fig 2B, left panel), which were significantly different (*P* < 0.01, Student’s paired *t*-test). The mean values of the Δangle under the R_on_L_on_/R_off_L_on_ and R_on_L_on_/R_off_L_off_ conditions were 3.6° ± 1.2° and 2.0° ± 0.8°, respectively (Fig 2B, right panel), which were also significantly different (*P* < 0.05, Student’s paired *t*-test).

### Experiment 2

**Fig 3:**
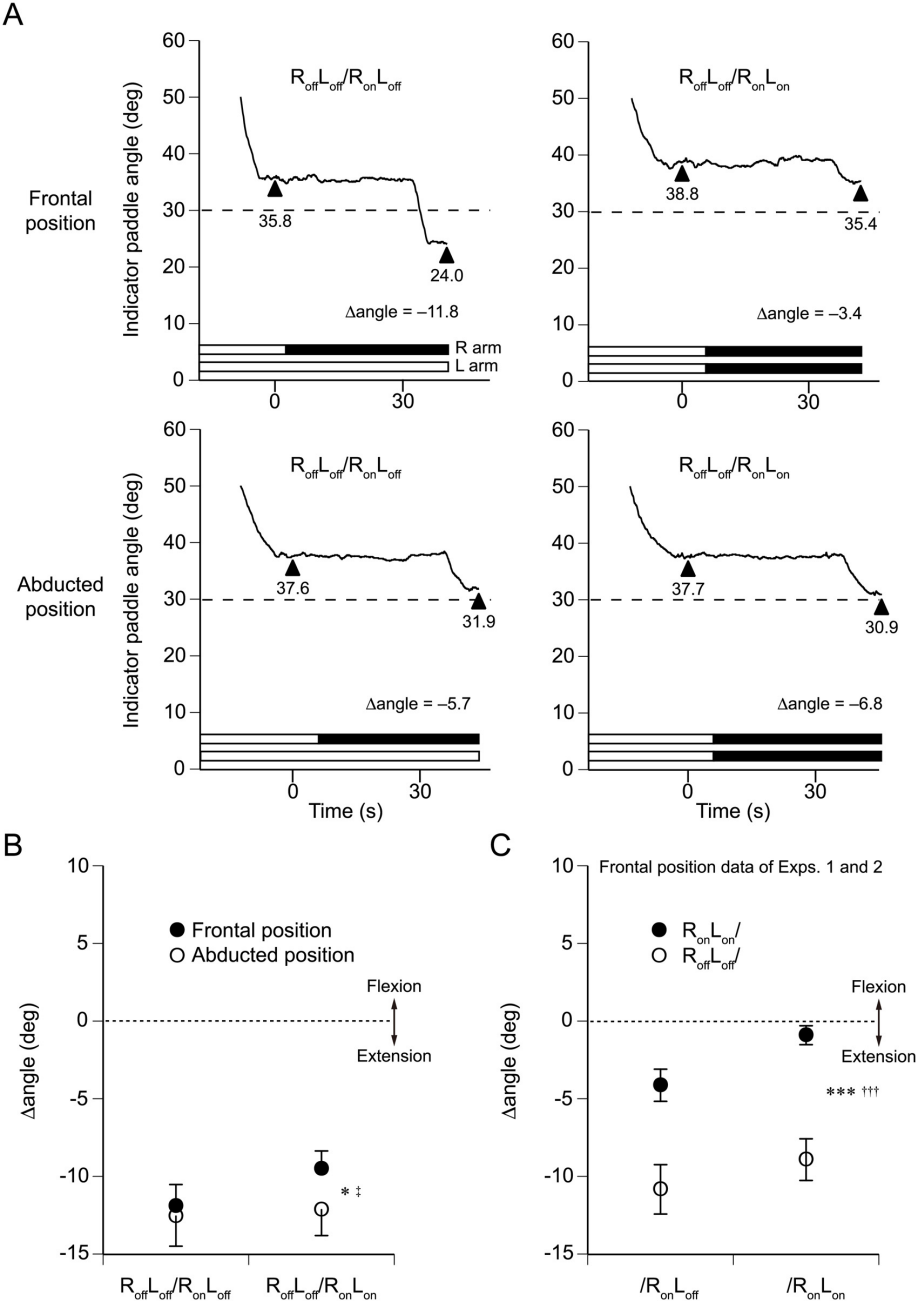
Effects of non-reference arm vibration and abduction on reference arm pointing errors. (A) Each panel represents a single arm pointing trial in a single participant in Experiment 2. The continuous line in each panel indicates the position of the indicator paddle. The position of the reference (right) arm was always in front of the body with a vertical angle of 30° (dashed lines). The left arm was placed in front of the body (upper panels), or it was abducted (lower panels). (B) Pooled data for the group (*n* = 10). A two-way ANOVA revealed a significant interaction between the position effect and the left arm vibration effect (^‡^ *P* < 0.05). The position effect was also significant (*P < 0.05). (C) Pooled data for the group of seven volunteers who participated in both Experiment 1 (R_on_L_on_/R_on_L_on_ and R_on_L_on_/R_on_L_off_ conditions) and Experiment 2 (R_off_L_off_/R_on_L_on_ and R_off_L_off_/R_on_L_off_ conditions with the left arm placed in front of the body). The left arm vibration effect was significant (***P < 0.001). The preceding bilateral vibration effect was also significant (^†††^ *P* < 0.001). However, no significant interaction was found between these effects. Values are means ± SEM.

Example traces for one participant are shown in Fig 3A. The upper two panels show the angle of the indicator paddle when both of the participant’s arms were aligned with one another in front of the body. Under the R_off_L_off_/R_on_L_off_ condition with the left arm placed in the frontal position (Fig 3A, upper left panel), this particular participant perceived that the unseen right arm was placed at angle of 35.8°. For the second pointing task, the right arm was vibrated, while the left arm was not. After a 30-s vibration period, this participant’s choice initially fluctuated but then the participant declared a match when the indicator paddle reached 24.0° (Fig 3A, upper left panel), with the resulting Δangle calculated at −11.8°. These results indicate that the participant perceived his vibrated reference arm to have extended 11.8°. However, when left arm vibration was added, the degree of the extension illusion elicited by the right arm vibration was less (Δangle = −3.4°, Fig 3A, upper right panel). To summarize the data generated in Experiment 2 to this point, as expected from Experiment 1, when both arms were aligned with one another in front of the body, the left arm vibration reduced the degree of the extension illusion elicited by the right arm vibration.

By contrast, when the left arm was placed in an abducted position, the left arm vibration had little effect on the degree of the extension illusion elicited by the right arm vibration (Fig 3A, lower panels). When the left arm vibration was off, the participant perceived his vibrated right arm to be moved into an extension (Δangle = −5.7°, Fig 3A, lower left panel). When the left arm vibration was added, the participant perceived his vibrated right arm to be moved into an extension to a similar degree (Δangle = −6.8°, Fig 3A, lower right panel).

The pooled data for the 10 participants showed results similar to those from the individuals (Fig 3B). With the left arm placed in front of the body (filled circles in Fig 3B), the right arm vibration produced larger angle changes when the left arm vibration was off (R_off_L_off_/R_on_L_off_, Δangle = −11.9° ± 1.4°) than when it was turned on (R_off_L_off_/R_on_L_on_, Δangle = −9.5° ± 1.2°). However, with the left arm placed at an abducted position (open circles in Fig 3B), the right arm vibration produced similar angle changes in the absence (R_off_L_off_/R_on_L_off_, Δangle = −12.6° ± 1.9°) and presence (R_off_L_off_/R_on_L_on_, Δangle = −12.1° ± 1.7°) of the left arm vibration. The Δangle was compared with two-way repeated-measures analysis of variance (ANOVA) to test for within-factor effects (the left arm position effect and the left arm vibration effect) and interactions between these effects (IBM SPSS Statistics 21). The existence of a significant interaction between the two effects (*F*_(1,9)_ = 8.9, ^‡^ *P* < 0.05) suggested that the left arm vibration effect on Δangle was more obvious when the left arm was placed in front of the body than when it was placed at an abducted position. The left arm position effect was also significant (*F*_(1,9)_ = 5.7, *P < 0.05). The mean values of the indicator paddle angle for the first pointing task were similar: 32.0° ± 1.7° (R_off_L_off_/R_on_L_off_, frontal position), 31.0° ± 1.8° (R_off_L_off_/R_on_L_on_, frontal position), 30.9° ± 1.8° (R_off_L_off_/R_on_L_off_, abducted position), and 31.3° ± 1.8° (R_off_L_off_/R_on_L_on_, abducted position).

We further analyzed data from seven volunteers who participated in both Experiments 1 and 2 to investigate whether vibrating both arms before the first pointing task alters the left arm vibration effect on Δangle (Fig 3C). We used data under four conditions: R_on_L_on_/R_on_L_on_, R_on_L_on_/R_on_L_off_, R_off_L_off_/R_on_L_on_, and R_off_L_off/_R_on_L_off_. The former two conditions were tested in Experiment 1. The latter two conditions were tested in Experiment 2, and we used Δangle collected when the left arm was placed in front of the body in Experiment 2 because Δangle was measured under R_on_L_on_/R_on_L_on_ and R_on_L_on_/R_on_L_off_ conditions with the left arm positioned in front of the body in Experiment 1. Two-way repeated-measures ANOVA revealed significant main effects (the left arm vibration effect, *F*_(1,6)_ = 66.2, ***P < 0.001; the preceding bilateral vibration effect, *F*_(1,6)_ = 50.1, ^†††^ *P* < 0.001). However, no significant interaction was found between these effects. The lack of this interaction suggests that the left arm vibration after the first pointing task alters Δangle to a similar extent regardless of vibrating both arms before the first pointing task.

### Experiment 3

In Experiment 2, when the left arm was placed at an abducted position, the left arm vibration had little effect on the vibration-induced extension illusion of the right arm. This result led to the next question: when one arm is placed in an abducted position, is the extent of the afferent signals from the abducted arm to the brain the same as when the arm placed is placed in front of the body? Throughout Experiment 3, the left arm was used as the reference arm, unlike in the previous two experiments. The participants were asked to specify the vertical position of the unseen left arm with the indicator paddle. The right arm remained stationary in front of the body at a vertical angle of 30°.

**Fig 4:**
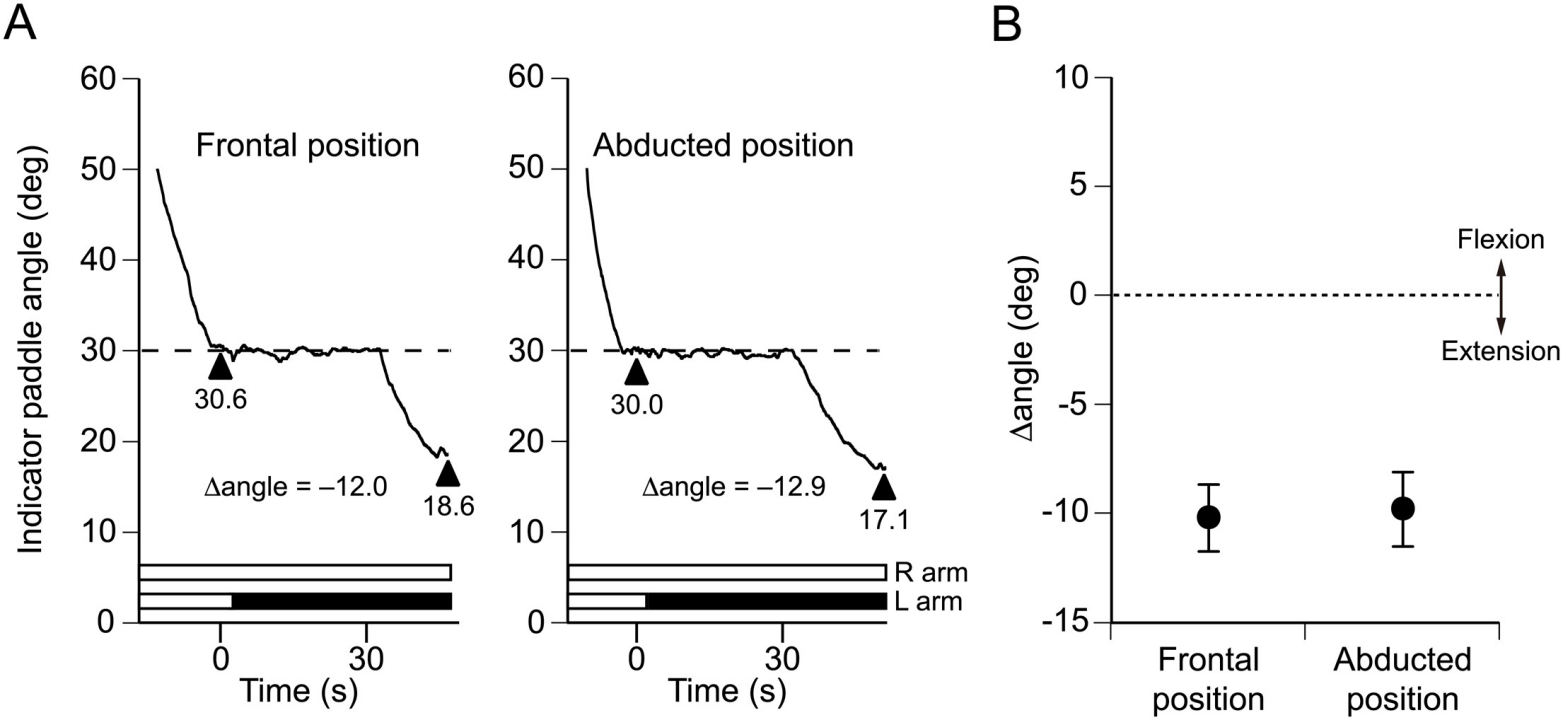
Effects of reference arm abduction on pointing errors in its own position. (A) Each panel shows the traces from a single arm pointing trial in a single representative participant obtained during Experiment 3. The reference arm, which was the left arm in this experiment, was placed either in front of the body or at an abducted position at a vertical angle of 30°. (B) Pooled data for 10 participants. There was no significant difference in the Δangle when the left arm was placed in the front or in an abducted position. Values are means ± SEM.

Traces from a representative participant are shown in Fig 4A. Before the start of left arm vibration, this participant perceived the vertical angle of his left arm in both positions with reasonable accuracy and the Δangle at both positions was similar (front position: 30.6°, Δangle –12.0°; abducted position: 30.0°, Δangle –12.9°). Pooled data for the 10 participants are shown in Fig 4B. The mean indicator paddle angles for the first pointing task were 29.2° ± 1.8° (frontal position) and 29.0° ± 1.9° (abducted position), suggesting that the participants were able to accurately identify the vertical positions of their left arms regardless of arm position. The lack of a significant difference in the Δangle with the left arm in front of the body (−10.2° ± 1.5°) or at an abducted position (−9.8° ± 1.7°) (*P* = 0.472, Student’s paired *t*-test) indicated that left arm vibration produced an extension illusion in the left arm to a similar degree regardless of whether the left arm was placed at an abducted position or in front of the body.

## Discussion

In the present study, we conducted three experiments to show that proprioceptive interactions occurred between the two arms in a one-arm pointing task. Although this task had been thought to be a one-arm proprioceptive test [6], our results indicated that there was proprioceptive input from both arms. In Experiment 1, proprioceptive illusions elicited by the vibration of the elbow flexors in one arm were reduced by increases in the muscle spindle signals from the other arm in this one-arm pointing task. In Experiment 2, this interaction occurred when the other arm was vibrated at a position aligned with the reference arm, but it did not occur when the other arm was vibrated at an abducted position. In Experiment 3, the extent of the proprioceptive illusion elicited by vibration in one arm was similar whether the arm was placed at an abducted position or in front of the body. This result suggested that the brain received increased proprioceptive input from a vibrated arm even when the arm was placed at an abducted position. In Experiment 2, the brain presumably received increased proprioceptive input from the abducted arm when it was vibrated. However, a comparison mechanism for the proprioceptive signals from two arms, which remains controversial [7], would not come into play here, likely because the brain considered the positions of the arms irrelevant.

We recently reported on a new aspect of the comparison mechanism for proprioceptive signals from two arms in an arm matching task [2], and this aspect is consistent with the results of the present Experiment 1 using an arm pointing task. We reported that elbow flexor vibration of the reference arm could not fully elicit the proprioceptive illusions of elbow extension in an arm matching task if elbow flexors of the other arm were simultaneously vibrated [2]. This implies a degree of interaction for the incoming proprioceptive information from the two arms in the one-arm matching task. If an interaction in spindle signals between the two arms does not occur, the degree of the extension illusion elicited by elbow flexor vibration of the reference arm would be the same in the presence and absence of elbow flexor vibration of the other arm. Our explanation for the reduced proprioceptive illusion in an arm matching task [2] is that elbow flexor vibration of the other arm reduces the relative increase in muscle spindle activity in the reference arm in response to reference arm vibration. Presumably, spindle signals from both arms are integrated in the brain, and a difference signal is produced by neuronal computation to determine the perceptual representation of the reference arm’s position. Once the brain determines this new perceptual position, which includes the interaction between the signals from both arms, the other arm, used as an indicator, must move toward that position. Thus, the other arm does not appear to move toward a position that is deduced using signals derived only from the reference arm.

For the pointing task, information from the tested arm must be compared with a stored model of the metric properties for the limb [6]. This is different for the matching and pointing tasks. Proske and Gandevia [6] stated in their review paper that “while proprioceptors provide information about position and movement of the limb, they are unable to signal the length of limb segments and therefore the absolute location of the limb in space.” Presumably, arm matching is performed on the basis of the spindle signals from the two arms and the interaction between them [2, 29]. For a two-arm matching task, one arm acts as a reference point, and knowing the absolute location of the two arms in space is not required for this task. However, if the position of one arm alters the position sense of the other arm in a matching task, similarly to the pointing task, the absolute location of the two arms may also be involved in the matching task. By contrast, in an arm pointing task, participants must specify the absolute location of the unseen arm in space, and an indicator proprioceptive signal is no longer available. Thus, locating body parts in space requires a combination of afferent information and stored representations of the body [30]. In the present study, our participants may also have accessed such a central map to describe the angle of their unseen arms with the indicator paddle. Inui et al. [26] reported that a central map of the hand is updated with time by changes in peripheral afferent input for an experimental phantom hand. Based on the results of our Experiment 1, it is conceivable that a central map of one arm changes in response to proprioceptive signals from the other arm. Presumably, the reference arm vibration distorts a central map of the reference arm, and the distorted map is further updated by vibration of the other arm. As a result, in the present study, the vibration-induced extension illusion of the reference arm was altered by vibration of the other arm in the arm-pointing task.

When the arms were aligned with one another in front of the body in our Experiment 1, the proprioceptive illusions elicited by vibrating the elbow flexors in one arm were altered by increases in the muscle spindle signals from the other arm. However, in Experiment 2, such an interaction in the position sense did not occur when the other arm was vibrated at an abducted position. By contrast, in Experiment 3, the vibration-induced extension illusion of one arm was maintained even when the arm was placed at an abducted position. Presumably, the brain was able to sense the incoming positional information from the abducted arm. In the arm-pointing task, the brain appears to ignore the incoming information from the other arm when it is abducted, while primarily referring to a central body map to determine the position of the reference arm. We previously speculated that the behavioral importance of the body’s position matching mechanism, based on the proprioceptive signals coming from the two arms, resides in the need for accurate alignment of the two arms to act as a single instrument [7]. Our present results support the implication that this mechanism comes into play when the brain judges that the positions of the arms are relevant.

The duration of vibration exceeded 30 s. Vibration lasting for more than 30 s may confound the interpretation of our results. Our previous study showed that 10-s vibration of the biceps had little post-effect on the forearm position sense in a two-arm matching task [13]. However, 30-s vibration induces long lasting alteration of proprioceptive perception [31]. In particular, Experiment 1 seems to be involved with the post-effect of vibration given before and during the first pointing task. Under R_on_L_on_/R_off_L_off_ conditions, the mean value of the indicator paddle angle for the first pointing task was 17.1° ± 1.2°. This value meant that vibration illusions occurred in the right arm because pointing angles ranged between approximately 30 ± 2°when neither arm was vibrated in Experiments 2 and 3. Then, the mean value of the Δangle under R_on_L_on_/R_off_L_off_ conditions was 2.0° ± 0.8°. This indicates that the participants still felt their right arm placed at an extended position at the time of the second pointing task, suggesting that effects of vibrating before the first pointing task lasted. Because the mean value of the Δangle was 3.6° ± 1.2° under R_on_L_on_/R_off_L_on_ conditions, left arm vibration after the first pointing task (/R_off_L_on_) may have counterbalanced the post-effect of vibrating before the first pointing task (R_on_L_on_). Therefore, pointing errors measured in the second pointing task do not involve only the effects of vibration being given at the exact moment; the errors likely included the post-effects. Furthermore, the more than 30-s vibration may have caused “proprioceptive drift” [32]. There are time-dependent changes in the perceived position of the reference arm in an arm matching task [29], leading to the possibility that such a proprioceptive drift is included in our results.

We have to acknowledge several limitations of the present study. We did not determine how close the arms had to be before the interaction was perceived. Specifying boundary conditions when the interaction converts to a positive result will be the subject of our next investigation. Tsay et al. [29] proposed that such a neural mechanism concerned with comparing signals from the two arms functions within a short range, with vertical angle limits of 10° in an arm matching task. Thus, it is conceivable that the interaction between the two arms found in the present study also occurs for limited arm positions.

We measured the vertical angle of the participants’ forearm but did not measure the angle between participants’ arm and forearm. The latter angle may be more adapted to quantify the illusory elbow extension than vertical angles. The same vertical angles between the horizontal line and participants’ forearms could induce different elbow extensions depending on the position of the participants’ arm and their bodies. Furthermore, illusion induction can vary depending on the contraction or lengthening of the muscle [16, 33].

We could also have added conditioning of elbow extensors. In the present study, elbow flexors were conditioned to place their muscle spindle responsiveness in a defined thixotropic state [27, 28]. However, Proske and his colleagues have shown recently that in a two-arm matching task the brain is concerned with the signal difference from the antagonist pair of each arm [28, 34]. Thus, spindle signals from unconditioned elbow extensors may have affected the perception of the elbow angle, even in the pointing task.

We used the same indicator starting position and movement direction for every trial. Visual feedback of the indicator paddle may have something to do with our results [35]. We cannot exclude this possibility for biases.

The proprioceptive interaction between the two arms occurred in a one-arm pointing task when the two arms were aligned with one another. When position sense is measured using the one-arm pointing task within a range where the two arms are aligned out of sight, the brain uses proprioceptive information from the two arms and refers to a central body map in determining the position of the unseen reference arm. Arm pointing tasks have been regarded as single limb tasks [6], but measuring position sense in one arm with a pointer includes influences of incoming information from the other arm when both arms are placed in front of the body and parallel to one another.
